# Efficacy and safety of bronchoscopic lung volume reduction therapy in patients with severe emphysema: a meta-analysis of randomized controlled trials

**DOI:** 10.18632/oncotarget.19352

**Published:** 2017-07-18

**Authors:** Yong Wang, Tian-Wen Lai, Feng Xu, Jie-Sen Zhou, Zhou-Yang Li, Xu-Chen Xu, Hai-Pin Chen, Song-Min Ying, Wen Li, Hua-Hao Shen, Zhi-Hua Chen

**Affiliations:** ^1^ Department of Respiratory and Critical Care Medicine, Second Affiliated Hospital of Zhejiang University School of Medicine, Hangzhou, China; ^2^ State Key Lab of Respiratory Disease, Guangzhou, China

**Keywords:** bronchoscopic lung volume reduction therapy, severe emphysema, meta-analysis, endobronchial coils, endobronchial valves

## Abstract

**BACKGROUND:**

Increasing randomized controlled trials (RCTs) indicate that bronchoscopic lung volume reduction (BLVR) is effective for severe emphysema. In this meta-analysis, we investigated the efficacy and safety of BLVR in patients with severe emphysema.

**METHODS:**

PubMed, Embase and the Cochrane Library and reference lists of related articles were searched, and RCTs that evaluated BLVR therapy VS conventional therapy were included. Meta-analysis was performed only when included RCTs ≥ 2 trials.

**RESULTS:**

In total, 3 RCTs for endobronchial coils, 6 RCTs for endobronchial valves (EBV) and 2 RCTs for intrabronchial valves (IBV) were included. Compared with conventional therapy, endobronchial coils showed better response in minimal clinically important difference (MCID) for forced expiratory volume in 1s (FEV1) (RR = 2.37, 95% CI = 1.61 – 3.48, *p* < 0.0001), for 6-min walk test (6MWT) (RR = 2.05, 95% CI = 1.18 – 3.53, p = 0.01), and for St. George's Respiratory Questionnaire (SGRQ) (RR = 2.32, 95% CI = 1.77 – 3.03, p < 0.00001). EBV therapy also reached clinically significant improvement in FEV1 (RR = 2.96, 95% CI = 1.49 – 5.87, *p* = 0.002), in 6MWT (RR = 2.90, 95% CI = 1.24 – 6.79, p = 0.01), and in SGRQ (RR = 1.53, 95% CI = 1.22 – 1.92, p = 0.0002). Both coils and EBV treatment achieved statistically significant absolute change in FEV1, 6MWT, and SGRQ from baseline, also accompanied by serious adverse effects. Furthermore, subgroup analysis showed there was no difference between homogeneous and heterogeneous emphysema in coils group. However, IBV group failed to show superior to conventional group.

**CONCLUSIONS:**

Current meta-analysis indicates that coils or EBV treatment could significantly improve pulmonary function, exercise capacity, and quality of life compared with conventional therapy. Coils treatment could be applied in homogeneous emphysema, but further trials are needed.

## INTRODUCTION

Chronic obstructive pulmonary disease (COPD) is responsible for around 6 percent of all deaths worldwide in 2012, and will be the third leading cause of death by 2020 [1, 2]. Although emerging different pharmacological treatments have significantly improved the lung function and exercise tolerance in mild or moderate COPD patients, few effective options are available for severe COPD [3]. Lung-volume-reduction surgery (LVRS), removing particularly damaged emphysema, allowing the relatively good lung to expand and work more efficiently, has been found to improve the quality of life and pulmonary functionfor patients with severe heterogeneous emphysema [4, 5]. However, due to the invasiveness and higher mortality associated with LVRS, it remains controversial to recommend patients for this surgery [5, 6].

Less invasive bronchoscopic lung volume reduction (BLVR) has achieved improvement in severe emphysema, including endobronchial coils, endobronchial valves, bronchial vapor ablation, lung sealants, and airway bypass [7, 8]. Recently, several randomized controlled trials (RCTs) have compared BLVR to medical therapy for advanced emphysema, and the results were encouraging, as confirmed in published meta-analyses [9–11]. However, previous meta-analyses pooled less RCTs and information, and more RCTs were published recently. Thus, to provide the latest and most convincing evidence, we aimed to identify and review RCTs which examined the roles and safety of BLVR in patients with emphysema.

## RESULTS

### Study characteristics

**Figure 1:**
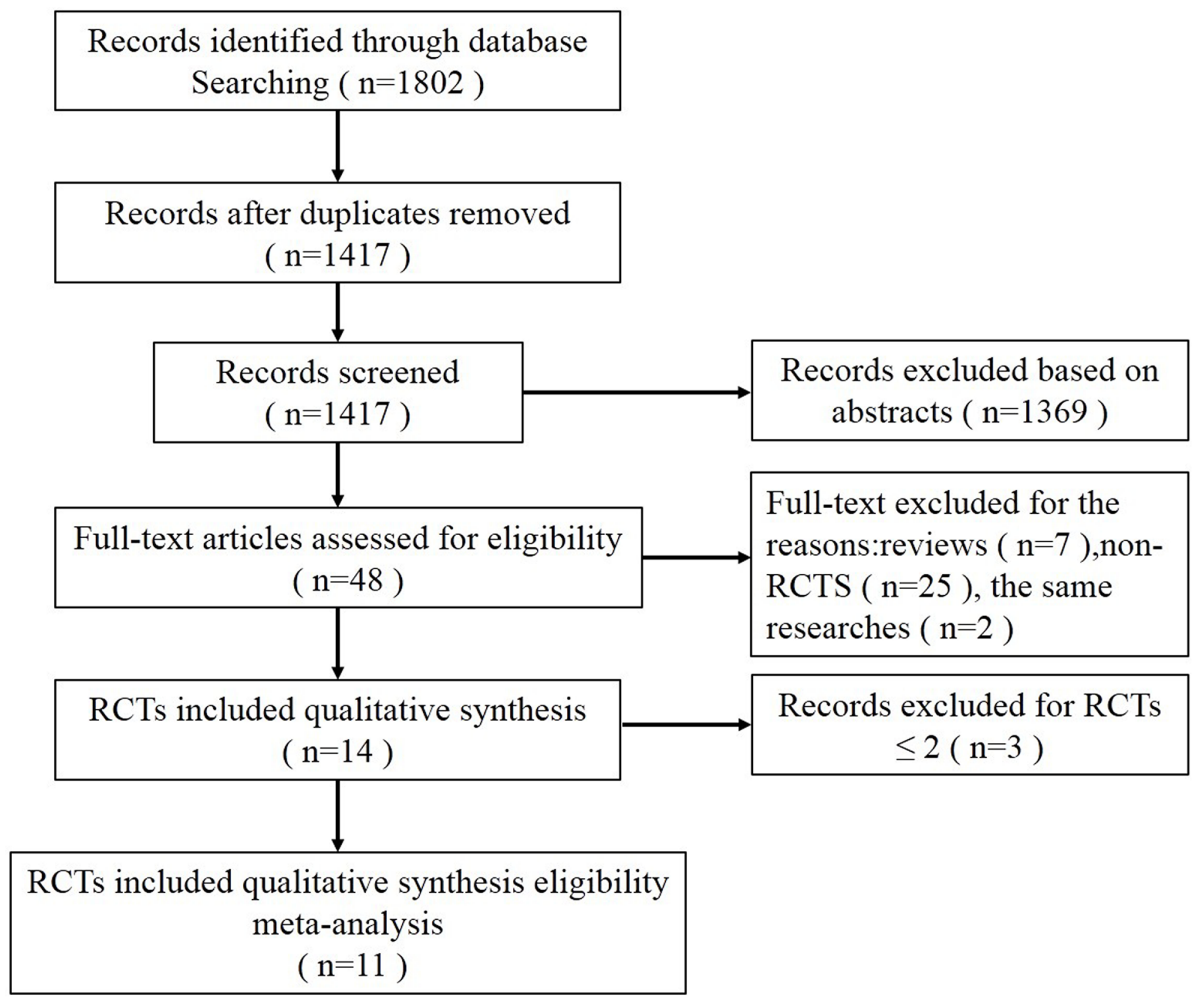
Flow diagram showing inclusion and exclusion of studies

The study flow diagram, including the reasons for exclusion of studies, is shown in Figure 1. A total of 1802 records were retrieved from the database search, of which 385 studies were excluded for duplicates. We excluded 1369 studies based on abstracts, and the remaining 48 full-text articles were assessed for eligibility. Of these, 34 studies were excluded for the following: reviews (n=7), non-RCTs (n=25), and the same researches (n=2). Also, 1 RCT for bronchial vapor ablation, 1 RCT for lung sealants, and 1 RCT for airway bypass were omitted [12–14]. Finally, 3 RCTs for endobronchial coils and 8 RCTs for valves were included in the meta-analysis [15–25]. Of the 8 studies for valves, 6 studies used endobronchial valves (EBV (Zephyr)) [15, 17, 18, 20, 21, 24] and 2 studies used intrabronchial valves (IBV (Spiration)) [16, 22]. The characteristics and inclusion and exclusion criteria of the included trials were presented in Tables 1 and 2. The study by Valipour [17] included the data from 416 patients with advanced emphysema across Europe VENT trial by Herth [20] and USA VENT trial by Sciurba [24].

**Table 1:** Baseline demographics and disease characteristics of the included RCTs

Study	Duration (months)	Sample size	Age (years), mean (SD)	FEV_1_ (% predicted) mean (SD)	6MWT (m) mean (SD)	SGRQ (points) mean (SD)	mMRC (points) mean (SD)	Goals in MCID (from baseline)
Sciurba 2010	3, 6, 12	EBV 220Control 101	EBV 65.3 (6.8)Control 64.9 (5.8)	EBV 30.0 (8.0)Control 30.0 (8.0))	EBV 333.9 (87.4 )Control 350.9 (83.2)	NR	NR	ΔFEV_1_ > 15%Δ6MWT > 15%ΔSGRQ (NR)ΔmMRC (NR)
Ninane2012	3	IBV 37Control 36	IBV 61.0 (7.0)Control 62.0 (6.0)	IBV 35.0 (10.0)Control 32.0 (7.0)	IBV 337.0 (106.0)Control 346.0 (123.0)	IBV 61.0 (11.0)Control 60.0 (13.0)	IBV 2.8 (0.7)Control 2.8 (0.9)	ΔFEV_1_ (NR)Δ6MWT (NR)ΔSGRQ ≥ 4 pointsΔmMRC (NR)
Herth2012	6, 12	EBV 111Control 60	EBV 59.7 (7.9)Control 60.4 (7.4)	EBV 29.0 (8.0)Control 30.0 (8.0)	EBV 341.0 (108.0)Control 360.0 (117.0)	EBV 59.0 (13.0)Control 56.0(18.0)	NR	ΔFEV_1_ ≥ 15%Δ6MWT ≥ 35metersΔSGRQ ≥ 4 pointsΔmMRC (NR)
Shah2013	3	Coils 23Control 23	Coils 62.0 (7.0)Control 65.3 (8.6)	Coils 27.2 (8.0)Control 28.9 (6.9)	Coils 293.7 (75.5)Control 346.2 (110.9)	Coils 65.2 (8.7)Control 53.1 (13.8)	Unclear	ΔFEV_1_ ≥ 10%Δ6MWT ≥ 26metersΔSGRQ ≥ 4 pointsΔmMRC (NR)
Valipour 2014	6	EBV 331Control 161	EBV 63.4 (7.7)Control 63.2 (6.9)	EBV 30.0 (8.0)Control 30.0 (8.0))	EBV 336.0 (95.0 )Control 356.0 (102.0)	EBV 54.4 (13.7)Control 52.8 (15.1)	EBV 1.9 (1.0)Control (NR)	ΔFEV_1_ ≥ 12%Δ6MWT ≥ 26metersΔSGRQ ≥ 4 pointsΔmMRC ≥ 1 point
Wood2014	6	IBV 142Control 135	IBV 64.7 (6.3)Control 64.8 (6.1)	IBV 29.8 (7.5)Control 29.7 (7.9)	IBV 314.1 (88.6)Control 308.6 (81.6)	IBV 54.8 (15.5)Control 57.1 (15.2)	IBV 2.7 (0.7)Control 2.7 (0.7)	ΔFEV_1_ (NR)Δ6MWT (NR)ΔSGRQ ≥ 4 pointsΔmMRC (NR)
Davey2015	3	EBV 25Control 25	EBV 62.3 (7.0)Control 63.3 (7.9)	EBV 31.6 (10.2)Control 31.8 (10.5)	EBV 342.0 (94.0)Control 334.0 (81.0)	EBV 67.8 (13.2)Control 70.7 (12.5)	EBV 4.0 (1.0)Control 4.0 (1.0)	ΔFEV_1_ ≥ 15%Δ6MWT ≥ 26metersΔSGRQ ≥ 4 pointsΔmMRC (NR)
Klooster 2015	6	EBV 34Control 34	EBV 58.0 (10.0)Control 59.0 (8.0)	EBV 29.0 (7.0)Control 29.0 (8.0)	EBV 372.0 (90.0)Control 377.0 (84.0)	EBV 59.1 (13.7)Control 59.3 (11.6)	EBV 2.7 (0.8)Control 2.7 (0.6)	ΔFEV_1_ ≥ 10%Δ6MWT ≥ 26metersΔSGRQ ≥ 4 pointsΔmMRC (NR)
Deslée2016	6, 12	Coils 50Control 50	Coils 62.1 (8.3)Control 61.9 (7.3)	Coils 25.7 (7.5)Control 27.4 (6.2)	Coils 300.0 (112.0)Control 326.0 (121.0)	Coils 60.8 (12.8)Control 57.1 (14.1)	Unclear	ΔFEV_1_ (NR)Δ6MWT ≥ 54metersΔSGRQ (NR)ΔmMRC (NR)
Sciurba2016	12	Coils 158Control 157	Coils 63.4 (8.1)Control 64.3 (7.8)	Coils 25.7 (6.3)Control 26.3 (6.7)	Coils 312.0 (79.1)Control 302.7 (79.3)	Coils 60.1 (12.8)Control 57.4 (14.8)	Unclear	ΔFEV_1_ ≥ 10%Δ6MWT ≥ 25metersΔSGRQ ≥ 4 pointsΔmMRC (NR)
Valipour2016	3	EBV 43Control 50	EBV 64.3 (6.3)Control 63.2 (6.0)	EBV 28.4 (6.3)Control 29.9 (6.6)	EBV 308.0 (91.0)Control 328.0 (93.0)	EBV 63.2 (13.7)Control 59.3 (15.6)	EBV 2.7 (0.8)Control 2.4 (1.0)	ΔFEV_1_ ≥ 12%Δ6MWT ≥ 26metersΔSGRQ ≥ 4 pointsΔmMRC ≥ 1 point

RCT = randomized controlled trial, Coils = endobronchial coils, EBV = endobronchial valves (Zephyr), IBV = intrabronchial valves (Spiration), MCID = minimal clinically important difference, FEV_1_ = forced expiratory volume in 1s, 6-min walk test (6MWT), SGRQ = St. George's Respiratory Questionnaire, mMRC = modified Medical Research Council, NR = not reported.

**Table 2:** Inclusion and exclusion criteria

Study	Major inclusion criteria	Major exclusion criteria
Sciurba 2010	Aged 40 to 75 years; Heterogeneous emphysema; 15 predicted < FEV_1_< 45% predicted; TLC >100% predicted; RV >150% predicted; PaCO_2_ < 50mm Hg and PaO_2_ >45mm Hg; 6MWT ≥140 m.	DLCO < 20% predicted; Giant bulla or α1-antitrypsin deficiency; Thoracotomy, Excessive sputum; Severe pulmonary hypertension; Active infection.
Ninane 2012	Aged 40 to 75 years; Predominantly upper lobe emphysema and severe dyspnea; FEV_1_ <45% predicted; TLC ≥100% predicted and RV ≥150% predicted; 6MWT ≥140 m.	DLCO < 20% predicted; Giant bulla or α1-antitrypsin deficiency; Severe pulmonary hypertension; Requirement for > 6 L O_2_ to keep saturation ≥ 90% with exercise; Thoracotomy.
Herth 2012	Similar to the study by Sciurba 2010.	Similar to the study by Sciurba 2010.
Shah 2013	Aged ≥35 years; Unilateral or bilateral emphysema; Homogeneous or heterogeneous emphysema; Post-bronchodilator FEV_1_ ≤ 45% predicted; TLC >100% predicted; mMRC dyspnoea score ≥2.	Change in FEV_1_ > 20% post-bronchodilator; DLCO < 20% predicted; Active infection, uncontrolled pulmonary hypertension; 6MWT≤ 140 m; Significant bronchiectasis; Giant bullae; Thoracotomy; Taking ≥ 20 mg prednisone daily.
Valipour 2014	Similar to the study by Sciurba 2010.	Similar to the study by Sciurba 2010.
Wood 2014	Aged 40 to 74 years; Predominantly upper lobe emphysema and severe dyspnea; FEV_1_ ≤ 45% predicted; TLC ≥ 100% predicted and RV ≥ 150% predicted; 6MWT ≥ 140 m.	FEV_1_ and DLCO < 20% predicted; PCO_2_ > 50 mm Hg, PaO_2_ < 45 mm Hg; Two or more hospitalizations for COPD exacerbation or respiratory infections in the past year; Excessive sputum; Taking ≥ 15 mg prednisone daily; Giant bulla, α1-antitrypsin deficiency; Severe pulmonary hypertension; Requirement for > 6 L O_2_ to keep saturation ≥ 90% with exercise; Thoracotomy.
Davey 2015	FEV_1_ ≤50% predicted; TLC ≥ 100% predicted and RV ≥ 150% predicted; 6MWT <450 m; mMRC dyspnoea score ≥3; Heterogeneous emphysema and intact adjacent interlobar fissures.	Excessive sputum; Lower limits for lung function were not otherwise formally defined but patients were excluded if they were considered clinically to be too restricted or frail to undergo bronchoscopy or to tolerate a pneumothorax.
Klooster 2015	Aged ≥ 35 years; Post-bronchodilator FEV_1_ ≤ 60% predicted, TLC ≥ 100% predicted and RV ≥ 150% predicted; mMRC dyspnoea score ≥ 1; Complete fissure between the target lobe and the adjacent lobe.	Collateral ventilation in the target lobe and failure to achieve lobar occlusion with endobronchial valves.
Deslée 2016	Bilateral emphysema; Post-bronchodilator FEV_1_ ≤ 50% predicted; TLC ≥ 100% predicted and RV ≥ 220% predicted; mMRC dyspnoea score ≥ 2.	Post-bronchodilator FEV_1_ < 15% predicted; Post-bronchodilator change in FEV_1_ > 20%; Severe recurrent respiratory infections requiring more than 2 hospitalization stays in the past year; Severe pulmonary hypertension; Unable to perform a 6MWT in room air; Giant bulla; Homogeneous emphysema; Significant bronchiectasis; Thoracotomy.
Sciurba 2016	Aged ≥35 years; Bilateral emphysema; post-bronchodilator FEV1 ≤ 45% predicted; TLC ≥ 100% predicted and RV ≥ 175% predicted; mMRC dyspnoea score ≥ 2.	Severe homogeneous emphysema; Post-bronchodilator change in FEV_1_ >20%; DLCO <20% predicted; PaCO_2_ >55 mm Hg, PaO_2_ <45 mm Hg; Recurrent significant respiratory infections in the past year; Severe pulmonary hypertension; 6MWT≤ 140 m; Significant bronchiectasis; Giant bulla or α1-antitrypsin deficiency; Thoracotomy; Taking >20 mg prednisone daily.
Valipour2016	Aged ≥40 years; Homogeneous emphysema; 15 % predicted ≤ FEV_1_≤ 45 % predicted ;TLC > 100% predicted, RV ≥ 200% predicted; 6MWT > 150 m; Collateral ventilation negative target lobe	Active pulmonary infection and more than 3 exacerbations with hospitalizations in the past year; Severe pulmonary hypertension; α1-antitrypsin deficiency; excessive sputum; PaCO_2_ > 55 mm Hg; Taking > 25mg Prednisolone daily; Giant bulla or α1-antitrypsin deficiency; Thoracotomy.

FEV_1_ = forced expiratory volume in 1s, TLC = total lung capacity, RV = residual volume, 6MWT = 6-min walk test, DLCO = carbon monoxide diffusing capacity, mMRC= modified Medical Research Council.

### Risk of bias

**Figure 2:**
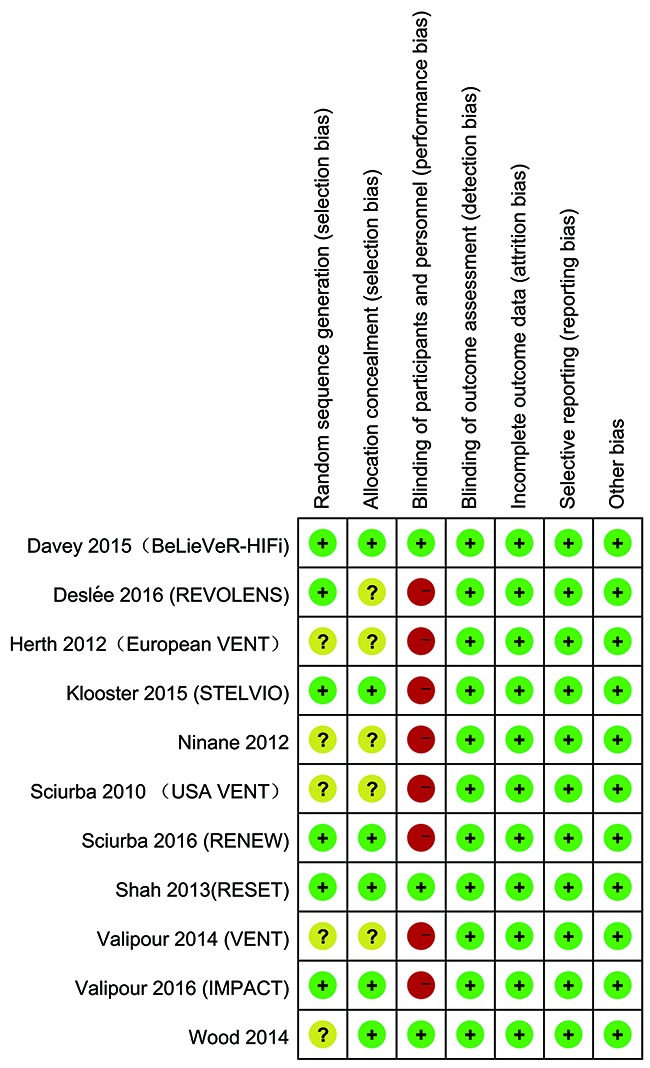
Risk of bias summary

The assessment of risk of bias was summarized in Figure 2. Six RCTs were judged to be an unclear risk of selecting bias, and eight RCTs generated high risk of bias in performance bias. It is very difficult in implementing a sham procedure in blinding of patients and clinicians in BLVR and may have influenced outcomes.

### Primary outcomes: responder analysis

**Figure 3:**
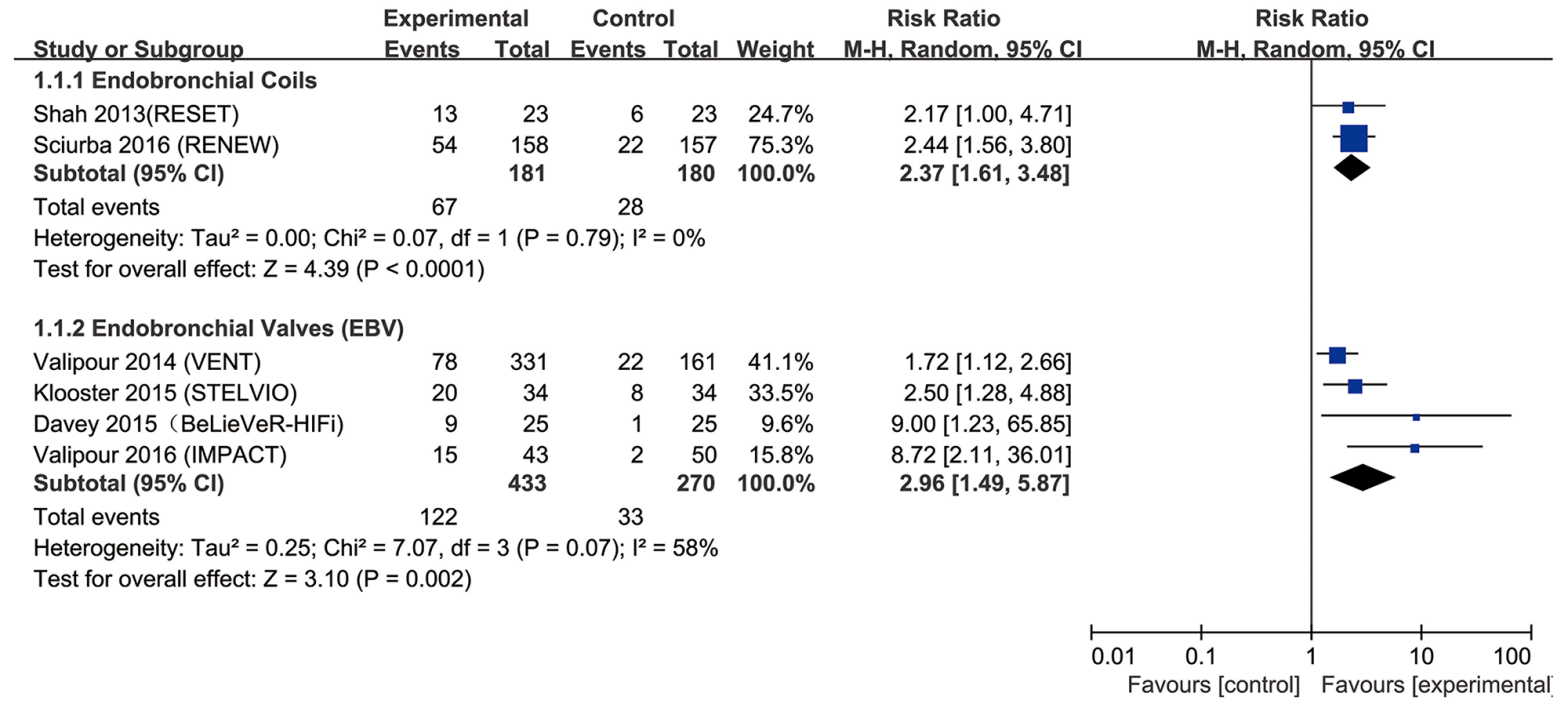
Effect of bronchoscopic lung volume reduction (BLVR) therapy on forced expiratory volume in 1s (FEV1) in patients with severe emphysema The term “Events” refers to the number of patients who reached MCID, and “Total” refers to the number of total patients. Risk ratios for each trial are represented by the squares, and the horizontal line crossing the square represents the 95% confidence interval (CI). The diamonds represent the estimated overall effect based on the meta-analysis random effect of the trials.

**Figure 4:**
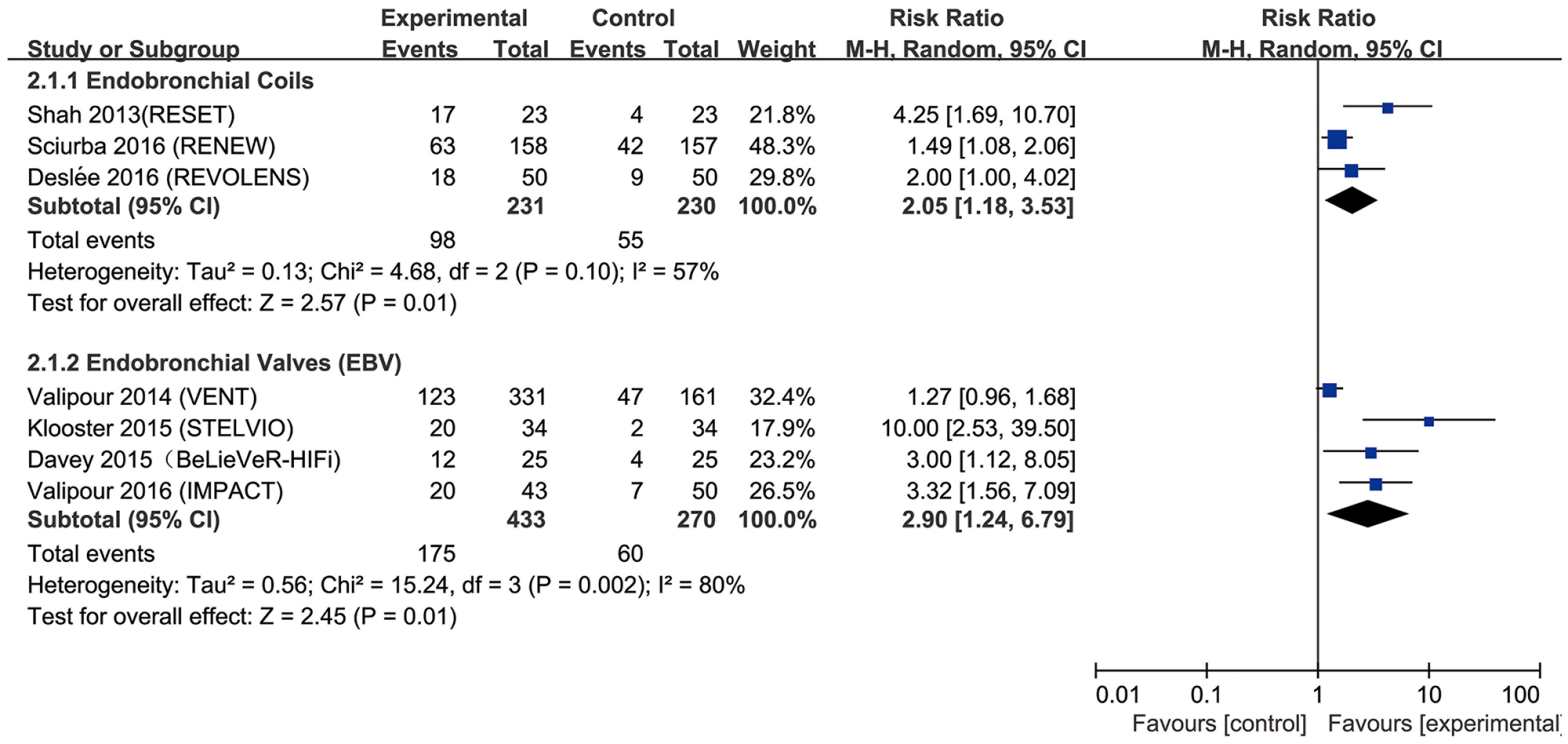
Effect of bronchoscopic lung volume reduction (BLVR) therapy on 6-min walk test (6MWT) in patients with severe emphysema The term “Events” refers to the number of patients who reached MCID, and “Total” refers to the number of total patients. Risk ratios for each trial are represented by the squares, and the horizontal line crossing the square represents the 95% confidence interval (CI). The diamonds represent the estimated overall effect based on the meta-analysis random effect of the trials.

**Figure 5:**
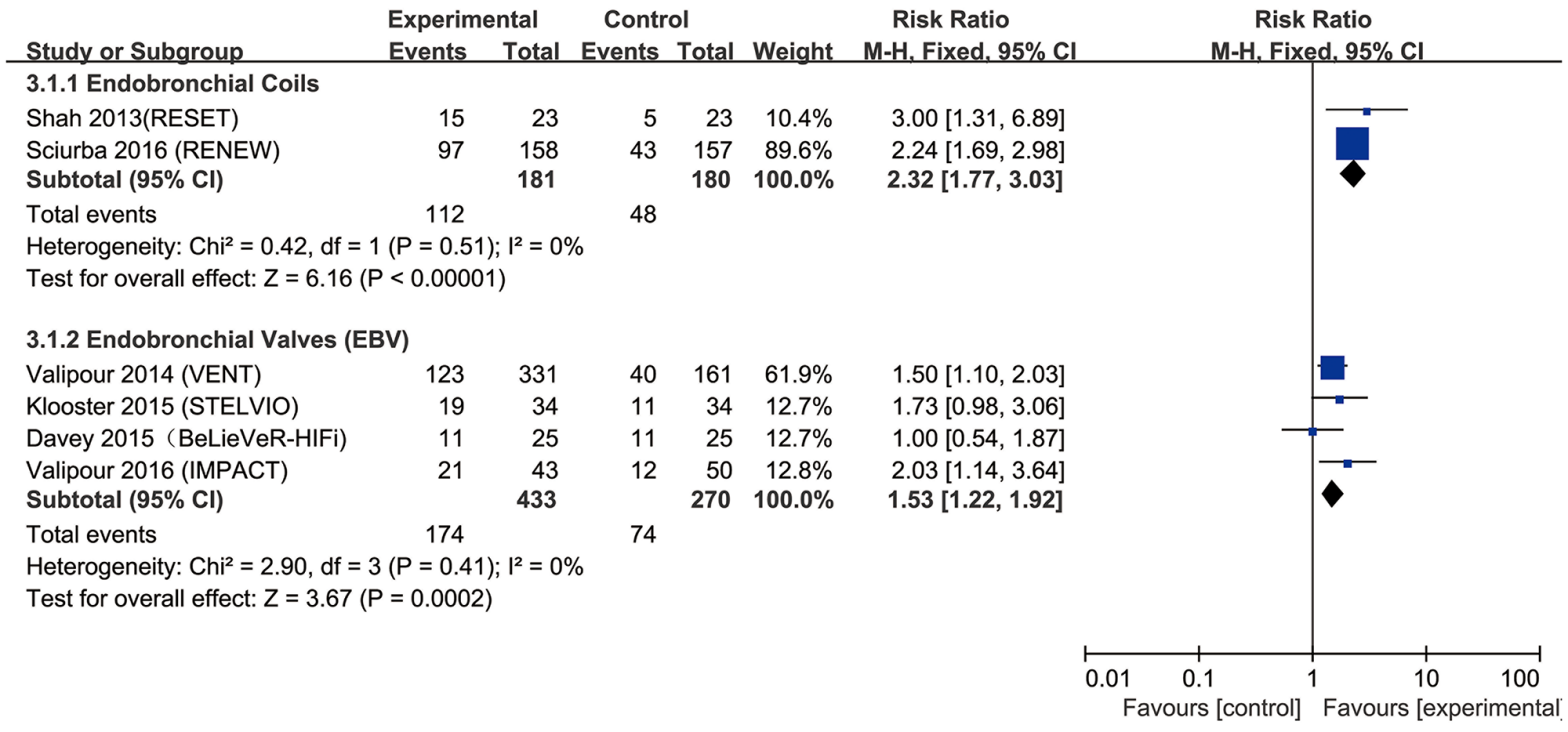
Effect of bronchoscopic lung volume reduction (BLVR) on St. George's Respiratory Questionnaire (SGRQ) in patients with severe emphysema The term “Events” refers to the number of patients who reached MCID, and “Total” refers to the number of total patients. Risk ratios for each trial are represented by the squares, and the horizontal line crossing the square represents the 95% confidence interval (CI). The diamonds represent the estimated overall effect based on the meta-analysis fixed effect of the trials.

**Figure 6:**
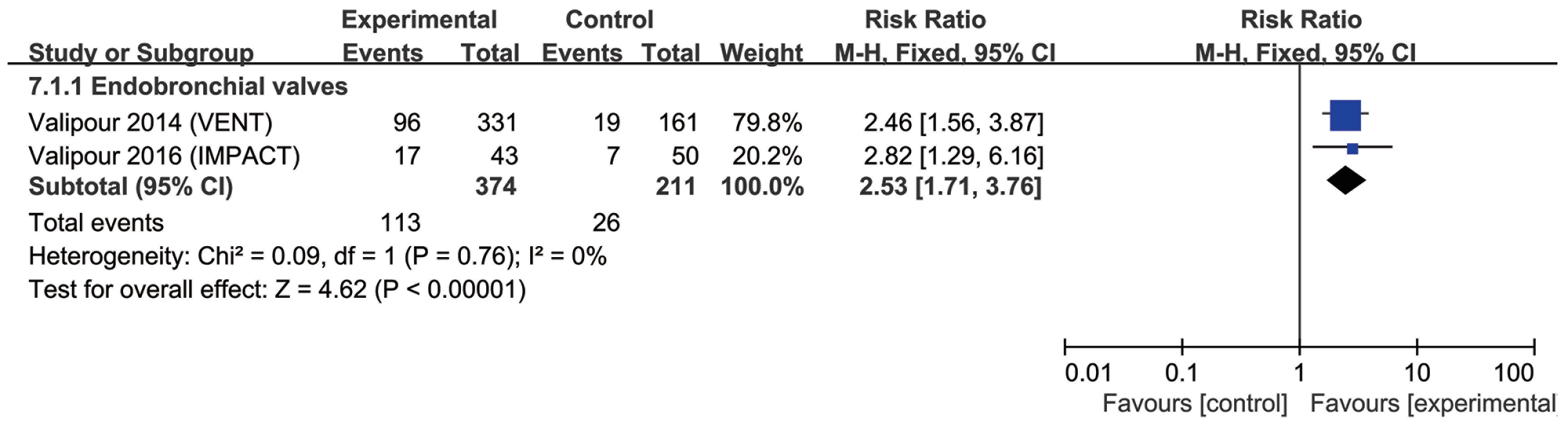
Effect of bronchoscopic lung volume reduction (BLVR) on modified Medical Research Council (mMRC) in patients with severe emphysema The term “Events” refers to the number of patients who reached MCID, and “Total” refers to the number of total patients. Risk ratios for each trial are represented by the squares, and the horizontal line crossing the square represents the 95% confidence interval (CI). The diamonds represent the estimated overall effect based on the meta-analysis fixed effect of the trials.

The most popular tools to assess lung function, exercise capacity and quality of life are forced expiratory volume in 1s (FEV_1_), 6-min walk test (6MWT) and St. George's Respiratory Questionnaire (SGRQ), which were used in almost all included studies. The modified Medical Research Council (mMRC) dyspnea scale was also used to assess quality of life. In the present study, endobronchial coils achieved a clinically significant improvement in FEV_1_ (risk ratio (RR) = 2.37, 95% confidence interval (CI) = 1.61 – 3.48, *p* <0.0001, I^2^= 0%), in 6MWT (RR = 2.05, 95% CI = 1.18 –3.53, *p* = 0.01, I^2^ = 57%), and in SGRQ (RR = 2.32, 95% CI = 1.77 – 3.03, *p* < 0.00001, I^2^ = 0%), compared with control (Figures 3–5). Also, EBV treatment showed better response in minimal clinically important difference (MCID) for FEV_1_ (RR = 2.96, 95% CI = 1.49 – 5.87, *p* = 0.002, I^2^ = 58%), for 6MWT (RR = 2.90, 95% CI = 1.24 – 6.79, *p* = 0.01, I^2^ = 80%), for SGRQ (RR = 1.53, 95% CI = 1.22 – 1.92, p = 0.0002, I^2^ = 0%), as well as for mMRC (RR = 2.53, 95% CI = 1.71 – 3.76, *p* <0.00001, I^2^ = 0%) (Figures 3–6).

### Secondary outcomes: absolute change in FEV1, 6MWT, SGRQ and mMRC from baseline, and safety assessment

Data for the absolute change BLVR were available from 11 RCTs (Table 3). For the coils treatment, the pooled weighted mean differences (WMD) in ΔFEV_1_ was 7.31% (95% CI = 4.65 – 9.97, *p* < 0.00001, I^2^ = 0%), in Δ6MWT was 31.72m (95% CI = 4.95 – 58.49, *p* = 0.02, I^2^ = 71%), in ΔSGRQ was -9.16 points (95% CI =-11.64 – -6.68, *p* < 0.00001, I^2^ = 0%) and in mMRC was -0.36 point (95% CI = -0.69 – -0.03, p = 0.03, I ^2^ = 0%) changed from baseline, compared with conventional therapy. Similarly, EBV therapy was associated with significant improvement in ΔFEV_1_ (WMD = 11.44%, 95% CI = 6.11 – 16.77, *p* < 0.0001, I^2^ = 57%), in Δ6WMT (WMD = 33.86m, 95% CI = 11.54 – 56.19, *p* = 0.003, I^2^ = 76%), and in ΔSGRQ (WMD = -7.06 points, 95% CI = -10.71 – -3.41, *p =* 0.0001, I^2^ = 63%), in ΔmMRC (WMD = -0.35 point, 95% CI = -0.56 – -0.14, *p* = 0.0008, I^2^ = 30%). Of note, subgroup analysis showed that EBV treatment is more effective in patients with complete fissures. Furthermore, we compared the roles of coils treatment between heterogeneous and homogeneous emphysema, and no significant differences were detected. However, IBV group failed to show superior to conventional group.

**Table 3:** Meta-analysis of bronchoscopic lung volume reduction (BLVR) therapy for severe emphysema

Study group or subgroup	Outcomes	Heterogeneity		Pooled results	
		**P_h_**	**I^2^ (%)**	**Effect (95%CI)**	***P*** **value**
**Coils group VS conventional group**	ΔFEV_1_ (%)	0.38	0	WMD 7.31 (4.65 to 9.97)	*p < 0.00001*
	Δ6MWT (m)	0.03	71	WMD 31.72 (4.95 to 58.49)	*p = 0.02*
	ΔSGRQ (points)	0.86	0	WMD -9.16 (-11.64 to -6.68)	*p < 0.00001*
	ΔmMRC (points)	0.48	0	WMD -0.36 (-0.69 to -0.03)	*p = 0.03*
**Coils group between heterogeneous and homogeneous emphysema**	ΔFEV_1_ (%)	0.39	0	WMD 0.63 (-5.00 to 6.26)	*p = 0.83*
	Δ6MWT (m)	0.83	0	WMD 4.78 (-15.11 to 24.68)	*p = 0.64*
	ΔSGRQ (points)	0.93	0	WMD -1.25(-4.89 to 2.38)	*p = 0.50*
	ΔmMRC (points)	None	None	None	None
**EBV group VS conventional group**	ΔFEV_1_ (%)	0.05	57	WMD 11.44 (6.11 to 16.77)	*p < 0.0001*
	Δ6MWT (m)	0.003	76	WMD 33.86 (11.54 to 56.19)	*p = 0.003*
	ΔSGRQ (points)	0.03	63	WMD -7.06 (-10.71 to -3.41)	*p = 0.0001*
**EBV group VS conventional group (complete tissue or low collateral ventilation)**	ΔmMRC (points)	0.23	30	WMD -0.35 (-0.56 to -0.14)	*p = 0.0008*
	ΔFEV_1_ (%)	0.97	0	WMD 17.50 (11.86 to 23.13)	*p < 0.00001*
	Δ6MWT (m)	0.10	58	WMD 50.17 (25.04 to 75.29)	*p < 0.0001*
	ΔSGRQ (points)	0.16	42	WMD -8.55 (-12.83 to -4.26)	*p < 0.0001*
	ΔmMRC (points)	None	None	None	None
**IBV group VS conventional group**	ΔFEV_1_ (%)	None	None	None	None
	Δ6MWT (m)	0.48	0	WMD -18.77 (-35.27 to -2.28)	*p = 0.03*
	ΔSGRQ (points)	0.24	28	WMD 2.30 (-1.50 to 6.11)	*p = 0.24*
	ΔmMRC (points)	0.71	0	WMD -0.08 (-0.29 to 0.13)	p = 0.47

Coils = endobronchial coils, EBV = endobronchial valves (Zephyr), IBV = intrabronchial valve (Spiration), WMD = weighted mean difference, FEV_1_ = forced expiratory volume in 1s, 6MWT = 6-min walk test, SGRQ = St. George's Respiratory Questionnaire, mMRC = modified Medical Research Council, P_h_ = P values for heterogeneity of Q test, CI = confidence interval.

The usual severe adverse effects related were deaths, pneumonia, pneumothorax, hemoptysis, and COPD exacerbation required hospitalization. Table 4 suggested that the coils treatment did not show more significant adverse effect on deaths, COPD exacerbation with hospitalization, or hemoptysis, but had higher incidence of pneumonia or pneumothorax than conventional treatment.

**Table 4:** Meta-analysis of safety comparing bronchoscopic lung volume reduction (BLVR) with conventional therapy on the major complications

Study group	Outcomes	Heterogeneity		Pooled results	
		**P_h_**	**I^2^ (%)**	**Effect (95%CL)**	***P*** **value**
**Coils group VS conventional group**	Deaths	0.94	0	RR 1.27 (0.59 to 2.72)	*p =* 0.54
	COPD exacerbation with hospitalization	0.95	0	RR 1.29 (0.81 to 2.05)	*p =* 0.28
	Pneumonia	0.98	0	RR 4.42 (2.20 to 8.88)	*p < 0.00001*
	Pneumothorax	0.54	0	RR 8.17 (2.22 to 30.03)	*p = 0.002*
	Hemoptysis	0.61	0	RR 5.98 (0.73 to 49.25)	*p = 0.10*
**EBV group VS conventional group**	Deaths	0.71	0	RR 1.56 (0.47 to 5.18)	*p = 0.47*
	COPD exacerbation with hospitalization	0.53	0	RR 2.01 (1.19 to 3.40)	*p = 0.01*
	Pneumonia	0.73	0	RR 2.17 (0.86 to 5.49)	*p = 0.10*
	Pneumothorax	0.65	0	RR 9.65 (3.04 to 30.60)	*p = 0.0001*
	Hemoptysis	0.43	0	RR 6.42 (1.21 to 34.01)	*p = 0.03*
**IBV group VS conventional group**	Deaths	0.73	0	RR 4.78 (0.84 to 27.31)	*p = 0.08*

Coils = endobronchial coils, EBV = endobronchial valves (Zephyr), IBV = intrabronchial valve (Spiration), P_h_ = P values for heterogeneity of Q test, CI = confidence interval, RR = risk ratio.

For EBV, we detected no between-group difference in deaths and pneumonia, compared with conventional treatment. However, pneumothorax, hemoptysis, or COPD exacerbation occurred more frequently in the EBV group. For IBV, there is no significant difference on deaths with conventional group.

## DISCUSSION

COPD is a progressive disease characterised by the permanent hyperinflation and decreased elasticity of air spaces distal to the terminal bronchioles. BLVR consists of the steps of introducing a bronchoscope into the airway to a position close to the damaged lung and equilibrates air within the damaged section with atmospheric air, thus ultimately deflating the hyperinflation of target lung tissue. BLVR is of great importance in the treatment of severe emphysema, particularly for the patients who are unresponsive to medical therapy and do not meet the criteria for LVRS.

The major findings of this systematic review and meta-analysis can be summarized as follows: 1) Compared with conventional therapy, endobronchial coils or EBV treatment provided obvious clinical benefits, with significant improvement in exercise capacity, quality of life, and pulmonary function in patients with severe emphysema, while no significant improvement was detected in IBV treatment. In addition, the absolute change in mMRC, below the defined MCID, indicated that coils or EBV play a significant but moderate role in improving dyspnea. 2) Subgroup analysis regarding efficacy showed no difference between homogeneous and heterogeneous emphysema in coils group. 3) There was no difference in deaths in the coils group or EBV group VS the conventional care group, but other serious adverse events occurred more frequently in the coils or EBV group.

Differences between the previous and present meta-analyses should be noted. A meta-analysis by Iftikhar showed efficacy in BLVR group, while only pooled the mean change post-intervention [9]. After that, two meta-analyses regarding EBV concluded that EBV treatment was associated with superior efficacy compared with conventional treatments, but the conclusion was limited by only two RCTs included [10, 26]. Meanwhile, all of the above studies did not analyze the MCID, which indicates minimal clinical benefits. A more recent meta-analysis also showed the clinical benefits of coils or EBV treatment in patients with advanced emphysema, but the results were based on the mean change from baseline in treatment group, without the comparison with conventional treatment [11]. Furthermore, all the previous meta-analyses did not include new high-quality published data, and some pooled RCTs, observation, or case-report data together, probably threatening the authenticity of their findings.

The highlighted strength of our systematic review and meta-analysis were as follows: 1) the present study included new recently published data and all included studies were the randomized controlled trials, limiting the confounding by indication and selection bias. 2) The analysis focused on the comparative efficacy between the coils or valves treatment and conventional treatment. 3) We analyzed the efficacy of responder for MCID, and also the difference between heterogeneous and homogeneous emphysema in coils group, which were more comprehensive.

Endobronchial valves are placed to block air from entering selective pulmonary lobe that may improve lung function and exercise tolerance. Valves have two devices, EBV and IBV, the efficacy of which are different. In this analysis, compared with conventional treatment, EBV treatment provided, while IBV treatment failed to achieve, significant improvements in patients with severe emphysema. The current meta-analysis also revealed that EBV treatment led to clinically meaningful improvements in pulmonary function, quality of life, and exercise capacity. Nowadays, patients with complete fissures might be considered for treatment with EBV and our analysis also suggests that EBV could show better outcomes in patients with intact fissures. Some authors have suggested that a greater response to EBV treatment is associated with heterogeneous emphysema and EBV should be considered for patients with heterogeneous emphysema, but recently, IMPACT trial [15] demonstrated clinically meaningful benefits in selected patients with homogeneous emphysema without collateral ventilation. It should be noted that emphysema heterogeneity is quantified by visually scoring CT scans and there is no consensus for defining heterogeneity [27]. Further studies to investigate the effect of EBV treatment in homogeneous emphysema are still needed.

Two RCTs were included to compare intrabronchial valves with standard medical care. For the IBV, there was no significant difference in mortality with conventional care. However, IBV failed to show a direct effect on lung function and exercise capacity, which is associated with different approach of intrabronchial valves placement. Also, the two studies selected patients only with upper lobe predominant emphysema and did not aim at achieving lobar occlusion, which may affect the results. Although previous RCTs did not achieve meaningful results, the IBV treat remains a promising treatment for selected patients with low collateral ventilation and complete occlusion of a single lobe [16], and the other two RCTs (NCT01812447, NCT01812447) for heterogeneous emphysema are ongoing, which may alter the present results.

Shape-memory nitinol coils are bronchoscopically designated to induce parenchymal compression, enhance lung recoil, and thus improve ventilator function. Our data suggested that coils treatment showed clinically meaningful benefits and statistically absolute change in 6MWT, and SGRQ in patients with severe emphysema, compared with conventional therapy. However, FEV1 showed a statistically absolute improvement of 7.31%, below the defined MCID. The variance in the baseline level and ceiling effect may limit the response of the FEV1 measurement and the results needs to be interpreted with caution. Further subgroup analysis showed that there were no significant differences in absolute change of FEV1, 6MWT, and SGRQ between heterogeneous emphysema and homogeneous emphysema. Thus, the efficacy of coils treatment is independent of heterogeneous emphysema and could be applied in homogeneous emphysema. Of note, LVRS or EBV treatment for patients with homogenous emphysema is debatable, although good results were published [15, 28, 29]. So, coils treatment may be more suited to homogeneous emphysema.

Compare with conventional treatment, endobronchial coils or valves treatment did not increase the mortality rate. But coils was accompanied by higher rate of pneumonia, pneumothorax, and the increase in pneumothorax, hemoptysis, and COPD exacerbation were associated with the EBV. Most of serious complications occurred early and much less frequently afterwards, which could be cured by common therapy. Thus, due to the low morbidity rate and acceptable side effects, endobronchial coils or EBV treatment is more opted for severe emphysema than LVRS.

There was higher heterogeneity in outcomes especially in the EBV treatment. Sciurba *et al.* [24] have reported that emphysema heterogeneity and fissure completeness were associated with an enhanced response to EBV treatment. Different types emphysema included in EBV studies may cause high degree of heterogeneity. Other reasons for heterogeneity may be the variance in the measure and baseline characters, and high risk of bias in performance bias, and so on.

Unfortunately, bronchial vapor ablation, lung sealants and airway bypass had to be excluded for only 1 RCT included. The study by Come et al [14] showed that lung sealants were more efficacious in FEV1, 6WMT and SGRQ than conventional treatments, but accompanied with more adverse events that required hospitalization. Of note, early termination of this study, and subsequently the lower number of patients and short follow-up made the results less convincing. STEP-UP trial [13], comparing bronchial vapor ablation to standard medical treatments, selected patients with severe upper lobe-predominant emphysema. In this study, bronchial vapor ablation achieved clinically meaningful and statistically significant improvements in FEV1 and SGRQ at 6 months, with an acceptable safety profile. But, 6WMT could not be assessed for the lack of change in baseline numbers. EASE trial showed that airway bypass resulted in acute reduction in regional air trapping in patients with severe homogeneous emphysema, but failed to sustain long-term benefits. There was no significant between-group difference on adverse events.

There were also potential limitations that should be taken into consideration for this analysis. Firstly, the durations of follow-up in included trials were different and the results were varying, which may cause the fading of the effect or true differences. Meanwhile, the long-term data were scarce, and the assessments of most studies were limited to 12 months. Thus, the efficacy and safety of coils or valves on long-term lung function, exercise tolerance, and quality of life improvement are not certain. Moreover, some values were presented as MEAN (95% CI). We pooled theses values by converting mean (95% CI) to MEAN ± SD, and the follow-up term of some RCTs was variant, which might lead to high degree of heterogeneity and misleading conclusion. Thirdly, the random-effects modeling was used because of the significant heterogeneity in some analyses, which might affect the results of the present study.

Despite these limitations, this meta-analysis reinforced the results that endobronchial coils and EBV treatment were superior to conventional treatment in patients with severe emphysema, and did not increase deaths rate, but associated with some serious adverse effects. Further long-term follow up is needed to assess efficacy and safety on health outcomes.

## MATERIALS AND METHODS

### Search strategy

A comprehensive search was conducted including PubMed, Embase and the Cochrane Library. The following search strategies were used: “bronchoscopic lung volume reduction”, “BLVR”, “endobronchial coils”, “endobronchial coil”, “endobronchial valves”, “endobronchial valve”, “intrabronchial valves”, “intrabronchial valve”, “bronchial vapor ablation”, “vapor ablation”, “lung sealants”, “lung sealant”, or “airway bypass”. We reviewed the full-text articles designated for inclusion. In addition, we also manually searched the reference of the included studies and published reviews.

### Study selection

As observational studies are highly liable to confound by indication and selection bias, we only included studies that were randomized controlled trial (published in English), comparing BLVR to medical therapy, and the population was emphysema patients. Included studies should report adverse effects, and any or all of the following outcomes: (1) FEV1; (2) 6MWT; (3) SGRQ; (4) SGRQ. Meta-analyses were carried out only when included RCTs ≥ 2 trials.

### Assessment of risk of bias

Two authors independently assessed the risk of bias of RCTs based upon The Cochrane Collaboration tool [30]. The following domains were evaluated: selection bias, performance bias, detection bias, attrition bias, reporting bias and other bias. Studies were independently assessed by two reviewers, and were divided into three categories: (1) low risk of bias; (2) unclear risk of bias; (3) high risk of bias, for one or more key domains.

### Data extraction and statistical analysis

Data were extracted using a standard collection form. Information from each study including author names, year of publication, number of patients, intervention, control, outcomes, and adverse effect were extracted by two independent reviewers. If several studies reported the same patients, we chose the largest study to avoid the duplication. If some studies reported the results at different follow up time point, we chose the available data at the longest time point. Discrepancies were resolved through team consensus.

MCID is the smallest change in an outcome that a patient would benefit [31], and offers a threshold above which outcome is experienced as relevant by the patient, avoiding the problem of mere statistical significance. So, we took the proportion of patients who reached MCID as the primary outcome, and the MCID was determined as follows: ΔFEV1 ≥ 10% [32], Δ6MWT ≥ 26 meters [33], ΔSGRQ ≥ 4 points [34], and ΔmMRC ≥ 1 point [35]. Secondary outcome was an absolute change from baseline in FEV1, 6MWT, SGRQ and mMRC. Severe common adverse effects related were deaths, pneumonia, pneumothorax, hemoptysis, and COPD exacerbation required hospitalization.

Dichotomous outcomes data were compared by RR with 95% CI and WMD were also calculated for continuous outcomes. Heterogeneity was tested with the *I*^2^ statistic. *I*^2^ values of 50% ~ 75% or 75% ~ 100% were considered to have moderate or high heterogeneity, respectively. The fixed-effects modeling was used, but if heterogeneity was significant (*I*^2^ > 50%), the random-effects modeling was carried out [36]. All statistical analyses were performed by using RevMan version 5.3 (The Nordic Cochrane Center). *P*-values < 0.05 was considered statistically significant.

## Abbreviations

BLVR: bronchoscopic lung volume reduction
RCT: randomized controlled trial
EBV: endobronchial valves
IBV: intrabronchial valves
MCID: minimal clinically important difference
FEV1: forced expiratory volume in 1s
6MWT: 6-min walk test
SGRQ: St. George's Respiratory Questionnaire
COPD: chronic obstructive pulmonary disease
LVRS: lung-volume-reduction surgery
RR: risk ratio
CI: confidence interval
WMD: weighted mean differences
NR: not reported
P_h_: p values for heterogeneity of Q test
